# The Changes in Rats with Sciatic Nerve Crush Injury Supplemented with Evening Primrose Oil: Behavioural, Morphologic, and Morphometric Analysis

**DOI:** 10.1155/2017/3476407

**Published:** 2017-05-23

**Authors:** Danial Ramli, Izzuddin Aziz, Masro Mohamad, Dauda Abdulahi, Junedah Sanusi

**Affiliations:** ^1^Department of Anatomy, Faculty of Medicine, University of Malaya, Kuala Lumpur, Malaysia; ^2^Department of Pharmaceutical Sciences, Faculty of Pharmacy, Cyberjaya University College of Medical Sciences (CUCMS), Cyberjaya, Selangor, Malaysia

## Abstract

Nerve crush injuries are commonly used models for axonotmesis to examine peripheral nerve regeneration. As evening primrose oil (EPO) is rich in omega-6 essential fatty acid component and gamma-linolenic acid, studies have shown the potential role of EPO in myelination. Seventy-two healthy adult Sprague-Dawley rats were classified into three groups: normal group, control group, and experimental group. The result indicates that there was significant difference in toe-spreading reflex between the normal and the control groups (1.9 ± 0.031, *p* < 0.05) and the normal and the EPO groups (0.4 ± 0.031, *p* < 0.05) and significant difference between EPO and the control groups (1.5 ± 0.031, *p* < 0.05). Regeneration of axons and myelin in nerve fibre in the EPO-treated group developed better and faster than in the control group. In the control group, the shape of the axon was irregular with a thinner myelin sheath. In the experimental group, the shape of the axons, the thickness of the myelin sheath, and the diameter of the axons were almost the same as in the normal group. In conclusion, EPO supplementation may be beneficial as a therapeutic option for disturbances of nerve interaction.

## 1. Introduction

Peripheral nerve encompasses all the nerve trunks and branches which lie outside the central nervous system. When a peripheral nerve is injured, the muscles supplied by that nerve do not receive messages from the brain. Therefore, they become weakened or paralysed [1, 2]. Traffic collisions usually induce traumatic nerve injuries resulting from disruption of the intraneural circulation [3, 4]. This condition consequently induces demyelinization, remyelinization, axonal degeneration and axonal regeneration, focal, multifocal, or diffuse nerve fibre loss, and endoneural edema [4, 5]. Nerve regeneration is a complex phenomenon that has been gaining interest among scientists for many years. Many experimental studies have focused on treatment options to enhance the recovery process of injured peripheral nerves in the rat model. This includes the application of an electric field [6], extracts of various natural products, for example, the medicinal mushroom* Hericium erinaceus* [7], and surgical intervention, for example, nerve grafts and transplanting stem cells [8]. Many experimental studies have focused on treatment options to enhance the recovery process of injured peripheral nerves in the rat model. This includes the application of an electric field [6], surgical intervention, for example, nerve grafts and transplanting stem cells [8]. Furthermore, the transplantation of Schwann cells has also been shown to improve functional recovery and reduce histological deficits resulting from nerve crush injury [9]. Despite being the major producer of myelin in the peripheral nervous system, Schwann cells play an important role in promoting axonal regeneration by producing neurotrophic factors such as nerve growth factor (NGF) and ciliary neurotrophic factor (CNTF) [10]. Commercial drugs such as immunosuppressant and anti-inflammatory drugs may accelerate the rate of nerve regeneration following injury. However, they are associated with severe side effects such as high blood pressure, kidney problems, and liver disorders [11]. Therefore, it is important to search for natural products or substances and possible new drug treatments that could help improve nerve regeneration.

Evening primrose is a biennial herb originating from North America. Its botanical name is* Onoethera biennis.* The seeds of this herb are very valuable as they contain beneficial oil, evening primrose oil. The potential benefits of evening primrose oil, or EPO, are derived from the active ingredient called gamma-linolenic acid (GLA) (the major commercial source of GLA), which is an essential fatty acid. GLA is necessary for human health and may also be obtained from vegetable oils such as safflower oil* (Carthamus tinctorius)*, blackcurrant seed oil, and borage oil. EPO is one of the safest sources of GLA [12, 13]. EPO contains 8%–11% GLA and about 70% Linoleic acid such as Eicosanoid Acid (EA) and palmitic, stearic, and oleic acid [14]. Evening primrose is known as King's Cure-all [15]. This could possibly be due to its role in treating many diseases including atopic dermatitis, rheumatoid arthritis, mastalgia, and breast cysts [16]. In addition, EPO is also effective in controlling high blood pressure, assisting circulation, and maintaining healthy arteries, thereby preventing heart disease [17]. EPO has phytoestrogenic effects and is widely used as a complementary treatment in combating premenstrual tension and also as a postmenopausal treatment [18]. Certain studies have reported that consumption of EPO has been shown to prevent and rapidly reverse the nerve conduction velocity (NCV) of deficits found in the rat nerve. Diabetic animals and humans have a reduced ability to convert dietary linoleic acid into GLA. GLA and its metabolites are required for normal neuronal structure, function, and a normal microcirculation. A lack of GLA and its metabolites may play a major role in the development of neuropathy [19].

A study carried out where patients were given 360 mg GLA a day for a period of 6 months demonstrated that the patients who took GLA showed statistically significant improvement in neuropathy symptom scores after this time period [20]. The conclusion of the study was that GLA therapy might have a useful role in the prevention and treatment of distal diabetic polyneuropathy. Another study was conducted among patients taking 480 mg GLA a day for a one-year period concluded that GLA had a beneficial effect on the course of diabetic neuropathy [21]. In addition other studies have shown that motor and sensory conduction deficits were largely corrected in an EPO-treated reversal group, with the degree of amelioration compared to one-month diabetic control being 99% (*p* < 0.001) and 85% (*p* < 0.01), respectively [21]. Other researches have been done concerning peripheral nerve degeneration, as well as treatment and regeneration, from experimental studies in small animals, especially rats. This is because the speed of regeneration in these animals is fairly fast [22]. Sciatic nerve crush injuries are an ideal method of studying nerve injuries and regeneration. This is especially so when a therapeutic approach is under investigation, as it does not involve complete damage to the side of the nerve, for example, the epineurium, as is the case with section and repair [22]. The present study was conducted to observe the morphological changes to myelin and axons in rat sciatic nerve crush injuries using Toluidine Blue staining. The aim of this study is to determine the effect of EPO supplementation on the rate of peripheral nerve regeneration after sciatic nerve injury, through morphological and morphometric analysis of the injured nerve.

## 2. Materials and Methods

### 2.1. Animals and Surgical Procedures

In the present study, 72 healthy adult Sprague-Dawley rats that were about 8 weeks old (weighing 250 ± 50 g) were obtained from the animal house, Faculty of Medicine, University of Malaya. The care and procedures for animal experiments conformed to the guidelines of the Animal Care and Use Committee (ACUC) [Vot number ANA 14/07/2010/JS (R)], University of Malaya. The rats were classified into three groups.

*Group 1*. Normal group (*n* = 8): the rats did not undergo sciatic nerve crush injury and served as control for the histological study. The rats were fed with a conventional diet ad libitum.

*Group 2*. Control group (*n* = 32): the rats underwent sciatic nerve crush injury and were not supplemented with EPO. The rats were divided into four groups (1, 2, 3, and 4 weeks). Each group consisted of 8 rats. The rats were fed with a conventional diet ad libitum.

*Group 3.* Experimental group (*n* = 32): sciatic nerve crush injury was performed and the rats were supplemented with 6000 mg/day EPO. The rats were divided into four groups (1, 2, 3, and 4 weeks). Each group consisted of 8 rats. The rats were fed with a conventional diet ad libitum. The rats were anesthetized with a mixture of ketamine (100 mg/kg) and xylazine (10 mg/kg). The nerve crush procedure was performed on the right hind limb whereas the left side served as control. The site of the incision was shaved and the skin incised over a length of 2 cm along the proximal half of the line between the trochanter major and the knee joint. The right sciatic nerve was exposed through a gluteal muscle splitting incision, through which the overlying lateralis and biceps femoris muscles were separated without cutting the muscle fibres, using a pair of Watchmaker's forceps.

The visible sciatic nerve was crushed 1 cm proximal to the division of the sciatic nerve into the tibial and common peroneal nerves. The crush was achieved by applying 15 seconds of consistent pressure with a pair of sterile modified Watchmaker's forceps. A spacer was used at closure point to achieve moderate injury. The nerve was checked to ascertain that the epineurium was intact and that the nerve was completely crushed. This was done by raising the nerve slightly using a microprobe so that a clear area in the nerve was observed, which indicated a complete nerve crush. The skin incision was sutured and the rat was placed on a heating pad to keep it warm. Each rat received 1 ml of Lactated Ringer's solution subcutaneously and 0.4 mg/kg of Baytril intramuscularly. 10 mg/g of Chloramphenicol and 5 mg/g of Hydrocortisone Acetate ointment were applied to the incision area. The rats were monitored until they recovered from the anesthesia. After this, the rats were placed in their respective cages. Each rat was observed postoperatively to confirm complete crushing of the nerve shown by paralysis of the muscles to the toes and no spreading of the toes. Rats with movement in the toes, indicating incomplete nerve crush, were rejected from the group.

### 2.2. Histological and Morphometric Studies

The rats were sacrificed at different time points (7, 14, 21, and 28 days after operation). The point of the nerve crush was determined (1 cm before the bifurcation of the sciatic nerve) and the nerve was harvested for histological preparation. The sciatic nerve was dissected out and fixed in 4% Glutaraldehyde in Millonig's Phosphate Buffer for 24–72 hours, prior to the embedding process. The embedded sciatic nerve was serially cross-sectioned at a thickness of 50 *μ*m using a microtome. The sections of the sciatic nerve were stained with Toluidine Blue solution to quantitatively evaluate the regeneration of the sciatic nerve. The stained slides were mounted using DPX and observed under a light microscope equipped with a camera linked to a computer loaded with NIS-Element software to visualize the axons and myelin morphology of the sciatic nerve.

### 2.3. Dietary Supplementation

After the nerve crush procedure, the rats were fed according to the diet assigned for their groups for 4 weeks, during which behavioural testing was performed daily. All group were fed with standard rat chow. In addition, the experimental group were given EPO 6000 mg/day through an esophageal feeding tube starting from day 1 after nerve crush. The EPO administration is considered safe as no toxicity, carcinogenicity, or teratogenicity has been reported with high oral administration of EPO during clinical trial [23]. In addition, a higher dose was selected to determine if a higher dose than that used in Samsinah et al. (1999) would be better for nerve regeneration.

### 2.4. Functional Assessment

After surgery, the rats were inspected every single day. During these inspections, each rat was held by its tail above a surface and then lowered down onto it and carefully observed for a minute or two [23]. Activities were classified according to the toe-spreading reflex of the affected right hind limb: no spreading, minimal spreading, average spreading, and normal spreading. The toe-spreading reflex was elicited to measure the duration of complete recovery of the nerve. The behavioural observation was based on comparison of the reflex seen between the right side of the leg (where the nerve crush was performed) and the left side of the leg (which served as control). The first reading was at day 0 (after nerve crush injury) as shown in Table 1. The reflex was checked for 4 weeks. The scoring of the toe-spreading reflex was based on a 5-point scale as shown in Table 1.

### 2.5. Statistical Analysis

All the data were analyzed and compared between each group. Statistical significance was determined using one-way analysis of variance (ANOVA) and post hoc analysis (SPSS Version 20). Significant changes were determined using Tukey's test for multiple comparisons. Differences were considered to be significant at *p* < 0.05. The normality of all data distributions was checked using the Kolmogorov-Smirnov test.

## 3. Results

### 3.1. Histological Analysis of Myelin and Axon

**Figure 1:**
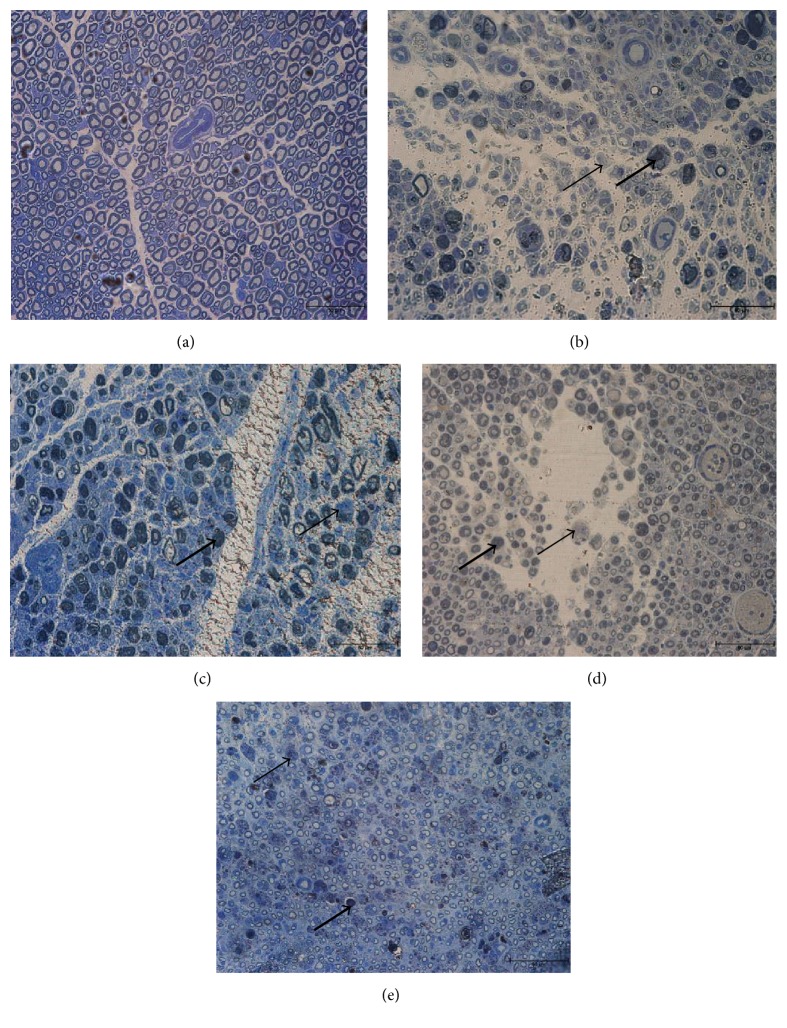
These photographs illustrated sections of sciatic nerve stained with Toluidine Blue at various time points. (a) Normal rat. (b) 7 days after operation. (c) 14 days after operation. (d) 21 days after operation. (e) 28 days after operation. Scale bar = 50 *μ*m* (arrow: macrophages, where the thick arrow indicates macrophage engulfing degenerating myelin sheath).*

The light microscopic sections of sciatic nerve stained with Toluidine Blue at various time points are shown in Figure 1, where Figure 1(a) represents a photomicrograph of a normal rat. It shows the normal distribution of axon size, number, and also myelin thickness. Few macrophages are visible in the normal group. From the histological study, it was clear that the axons in the normal group were consistently round in shape with a thick myelin sheath. The axons consisted of both myelinated and unmyelinated axons. A myelinated axon has a sheath of myelin layer around it while an unmyelinated axon does not. Besides that, the axon and myelin population was notably high. The axon/fibre diameter was also notably high.

When compared to the normal group, the control group depicts irregular axon shape with a thinner myelin sheath. The number of axon and myelin was lower than in the normal group. In this group, the diameter of axons was notably smaller than the normal and experimental group. At Day 7 after operation, as seen in Figure 1(b), there was a diffuse loss of large myelinated axons and both the axon number and myelin thickness had decreased tremendously. Macrophages (arrow in the figure) were visible and their number was greater. Starting from Day 14 up to Day 28 after injury, both axon number and myelin thickness increased with time.

**Figure 2:**
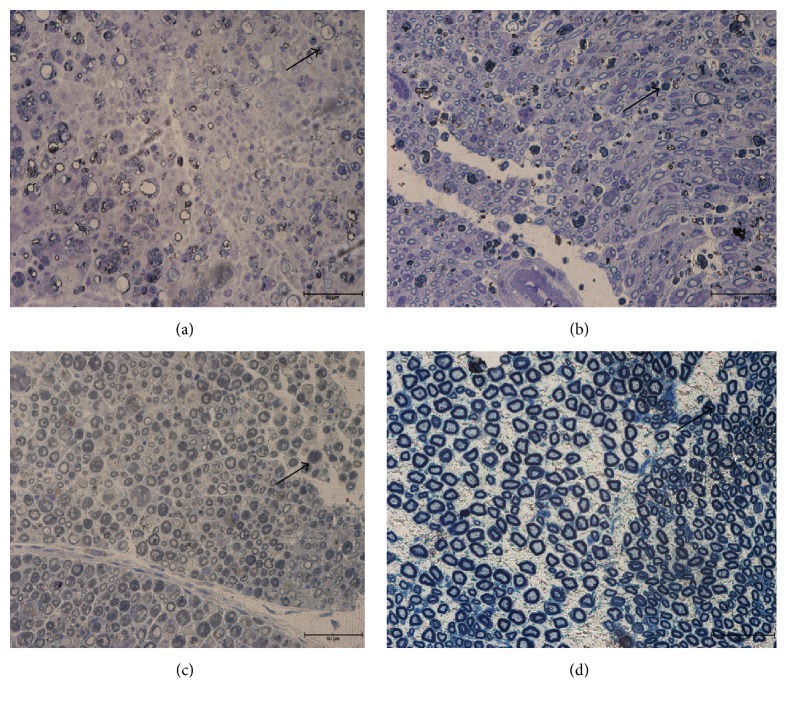
These photographs illustrated sections of sciatic nerve stained with Toluidine Blue at various time points. (a) 7 days after operation. (b) 14 days after operation. (c) 21 days after operation. (d) 28 days after operation (treated with EPO)* (arrow: macrophages).*

In the experimental group, axonal sprouting was noted with a population of smaller axons as early as Day 7 after operation (Figure 2(a)). Evidence of regeneration was noted by the appearance of a large number of macrophages populations (early nerve crush injury) which were swollen with the phagocytosis of degenerative debris, which strongly indicated that the nerve was still in the process of degeneration, while at Day 14 and Day 21 after operation, the population and size of the axon and myelin were increased over time (Figures 2(b) and 2(c)). In the experimental group, on Day 28 after operation (Figure 2(d)), the shape and diameter of the axons and also the thickness of the myelin sheath were similar to that of the normal group. The rats in the control group were still undergoing the regeneration process with the appearance of macrophages, when the rats in the experimental group had achieved complete recovery. However, in the control group, the axons were very small and there was no myelin sheath in nearly all of the axons in the nerve (refer to Figures 1(b), 1(c), and 1(d)). This indicates that the process of myelin sheath restoration was slow in the control group, as the experimental group had achieved complete recovery by the same time point.

### 3.2. Behavioural Analysis

**Figure 3:**
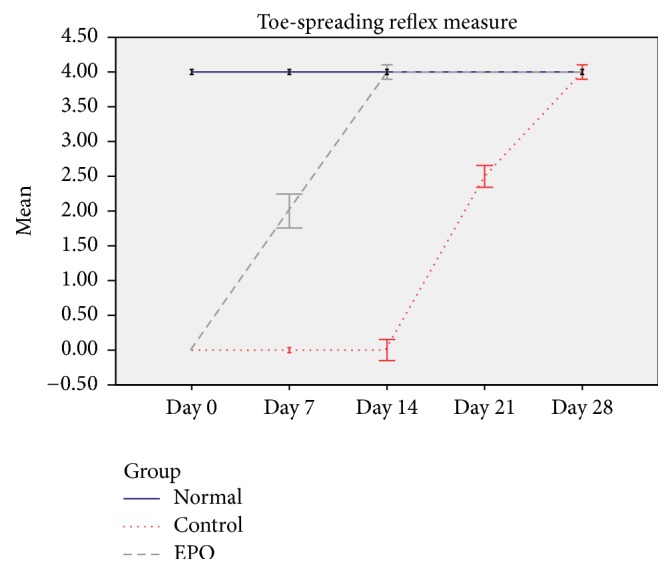
Graph of mean toe-spreading reflex score versus time.

The pairwise comparison tables and line graph of the mean score difference of toe-spreading reflex scores plotted for the normal, control, and experimental group are shown in Table 2 and Figure 3. The pairwise comparison reflects that there was significant difference in toe-spreading reflex between the normal and the control groups (1.9 ± 0.031, *p* < 0.05) and the normal and the EPO groups (0.4 ± 0.031, *p* < 0.05) and significant difference between EPO and the control groups (1.5 ± 0.031, *p* < 0.05) as shown in Table 2. The mean difference between the normal and EPO is evidently low, suggesting better TSR recovery rate. From the graph, a score of 4 (blue line) indicates the toe-spreading reflex of a normal uninjured hind limb while a score of 0 indicates an absence of reflex in any of the digits of the hind limb due to the immediate effect of sciatic nerve crush injury. Seven days after operation, the toe-spreading reflex in the control group (red line) was compared with the same reflex in the EPO-treated group (green line), the difference in the scores between these two groups was statistically significant (*p* < 0.05, two-tailed unpaired *t*-Test). In the control group, all rats showed some improvement (recovery process) in the toe-spreading reflex score 7 days after operation, but compared to the EPO-treated group it showed faster recovery as early as day 1 after operation. For the EPO-treated group, all the rats showed toe-spreading reflex score of 4 before surgery as they were in normal condition. After the sciatic nerve crush injury, the toe-spreading reflex score of the rats in control and experimental groups dropped as they lose their function. But, a week after nerve crush injury, the increased toe-spreading reflex score indicated that the rats have improved their function. The efficacy of EPO was seen when the toe-spreading reflex score for the experimental group was higher compared to those in the control group and the differences were significant; difference was seen at day 7 and 14 and 21 days after operation (*p* < 0.05, two-tailed unpaired *t*-Test).

### 3.3. Morphometric Evaluation Myelin and Axon

**Figure 4:**
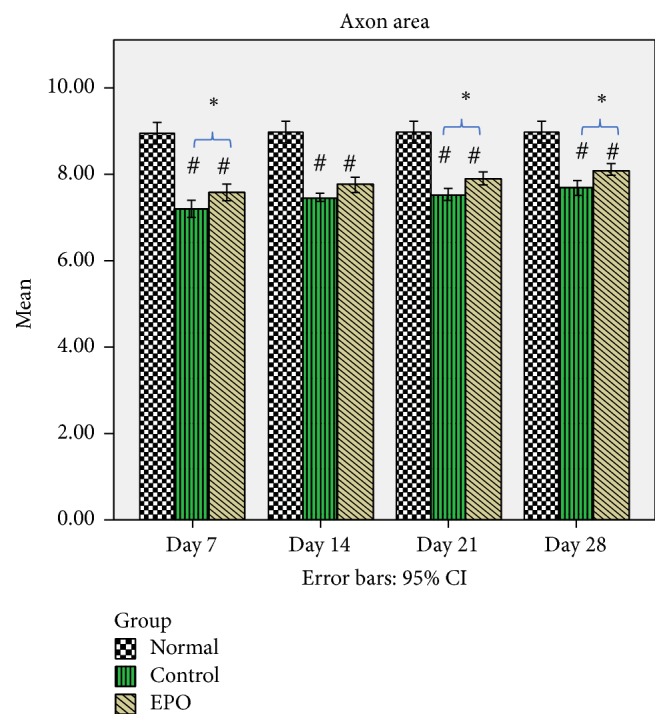
Mean axon area. The graph shows the value of the Mean Axon Area (*μ*m^2^) of the sciatic nerve with standard deviations (vertical bars). ^*∗*^*p* < 0.05*, one-way ANOVA when compared at the same time point between control and experimental groups. *^#^*p* < 0.05*, one-way ANOVA when normal group compared with control and experimental groups at each time point*.

The graph of the mean axon area of the sciatic nerve in the rats of the normal, control, and experimental groups is shown in Figure 4. From the data observed, after the nerve crush injury, the size of the axon area was significantly reduced (refer to control group at day 7 after operation, Figure 4) compared to the normal group, but after that the size of the axon area increased gradually over time. In the control group, it was found that at day 7 after operation there was a significantly higher percentage loss of the axon area (19.6%) compared to the experimental group, where it was only 15.3%. This shows that the EPO supplementation decreased axon area loss by 4.3%. In the control group at day 14 after operation it was found that there was a 16.8% loss of the axon area compared to the experimental group which was about 13.4%. At 21 days after operation 16% loss of the axon area was found compared to the experimental group which lost about 10.6%. While, in the control group, it was found that at day 28 after operation there was 14% loss of the axon area compared to the experimental group, where it was only 9.6% loss of axon area.

Thus, this data indicates that EPO supplementation may be related to successful regeneration growth following sciatic nerve crush injury in the rat.

**Figure 5:**
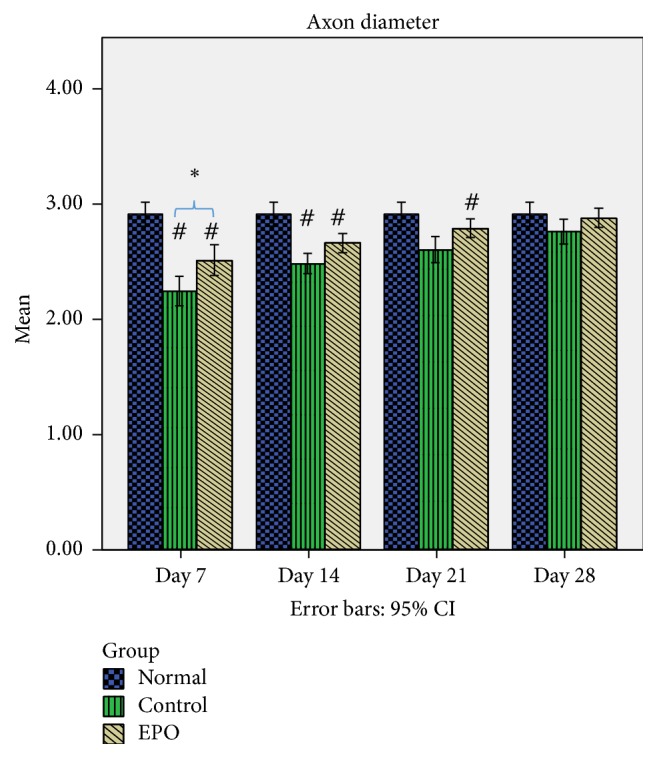
Mean axon diameter. The graph shows the value of the Mean Axon Diameter (*μ*m) of the sciatic nerve with standard deviations (vertical bars). ^*∗*^*p* < 0.05, one-way ANOVA when compared at the same time point between control and experimental groups. ^#^*p* < 0.05, one-way ANOVA when normal group is compared with control and experimental groups at each time point.

The mean axon diameter of the sciatic nerve in rats of the normal, control, and experimental groups is shown in Figure 5. From the data observed, after the nerve crush injury, the size of the axon diameter was completely reduced (refer to control group day 7 after operation) compared to the normal group but after that the size of the axon diameter increased gradually over time. In the control group, it was found that, at day 7 after operation, there was a significantly bigger percentage reduction of the axon diameter (23%) compared to the experimental group where it was only 13.7%. In the control group at days 14 and 21 after operation 14.8% and 10.7% loss of the axon diameter, respectively, were found compared to the experimental group which was about 8.6% and 4.1%, respectively, while, in the control group, it was found that at day 28 after operation there was 5.15% loss of the axon diameter compared to the experimental group, where it was only 1.03% loss of the axon diameter. Thus, this data indicates that EPO supplementation may be related to successful regeneration growth following sciatic nerve crush injury in the rat.

**Figure 6:**
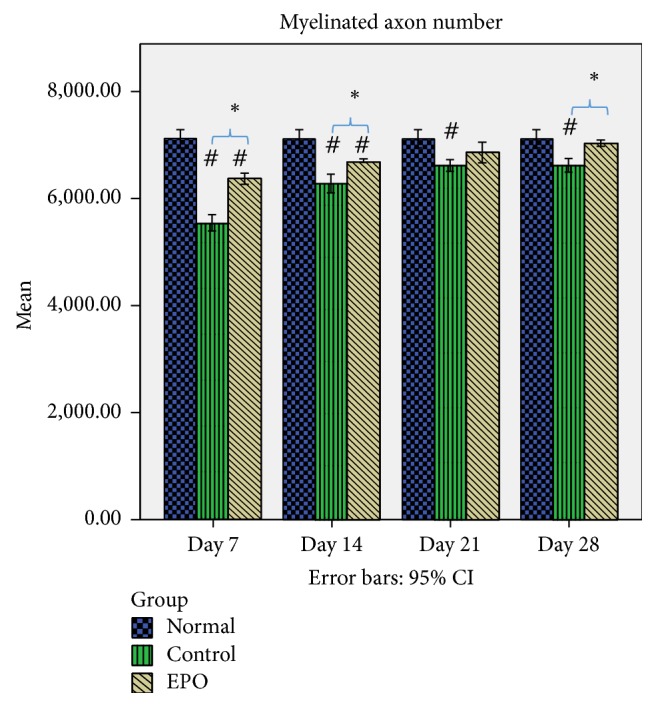
Mean myelinated axon number. The graph shows the value of the mean myelinated axon number of the sciatic nerve with standard deviations (vertical bars). ^*∗*^*p* < 0.05*, one-way ANOVA when compared at the same time point between control and experimental groups. *^#^*p* < 0.05*, one-way ANOVA when normal group is compared with control and experimental groups at each time point.*

Figure 6 shows the mean myelinated axon number which was 7116 for the control group, while for the rats at 7, 14, 21, and 28 days after operation it was 5535, 6281, 6624, and 6620, respectively, with significant differences between the control group and each of the experimental groups. This shows that, 28 days after the injury, the mean myelinated axon number was still significantly reduced/little compared to that of the control rats. There was a significant (*p* < 0.05) 22.2% loss of myelinated axons at day 7 after operation in the control group compared to the experimental group where it was only 10.4% at the same time point.

In the control group at days 14 and 21 after operation 11.7% and 6.91% loss of the myelinated axon number were found, respectively, compared to the experimental group which was about 5.8% and 3.5%, respectively, while, in the control group, it was found that at day 28 after operation there was 7% loss of the myelinated axons number compared to the experimental group, where it was only 1.2% loss of the myelinated axons number.

All the data show significant differences (*p* < 0.05) between the control group and experimental group at days 7, 14, and 28 after operation but no significant difference was found between the control and experimental group at day 21 after operation. Thus, this data indicates that EPO supplementation may be related to successful regeneration growth following sciatic nerve crush injury in the rat.

**Figure 7:**
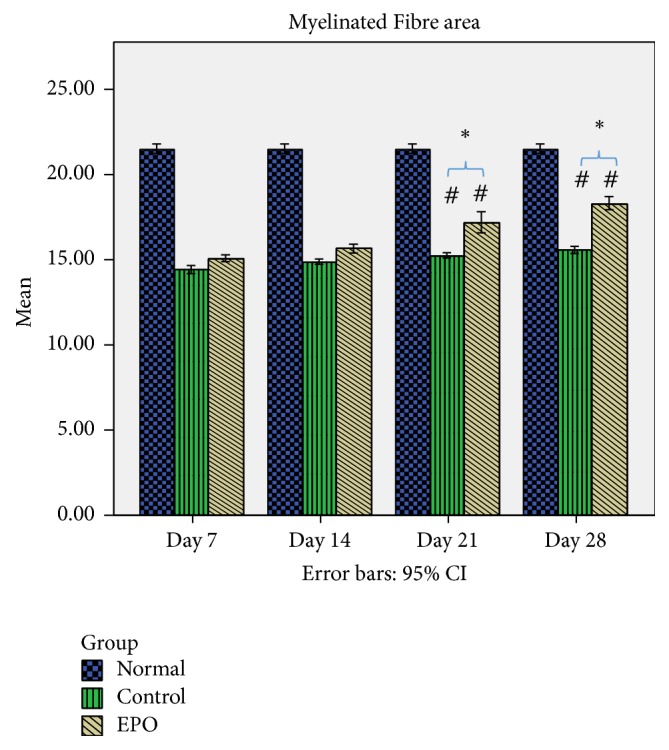
Mean myelinated fibre area. The graph shows the value of the mean myelinated fibre area of the sciatic nerve with standard deviations (vertical bars). ^*∗*^*p* < 0.05*, one-way ANOVA when compared at the same time point between control and experimental groups. *^#^*p* < 0.05*, one-way ANOVA when normal group is compared with control and experimental groups at each time point.*

Figure 7 shows the mean myelinated fibre area of the sciatic nerve in rats of the normal, control, and experimental groups. From the data observed, after the nerve crush injury, the mean myelinated fibre area was completely reduced (refer to control group day 7 after operation) compared to the normal group but after that it increased gradually over time. In the control group, it was found that at day 7 after operation there was a significantly higher percentage loss of the myelinated fibre area (33%) compared to the experimental group where it was only 29%. In the control groups at days 14 and 21 after operation 30.9% and 29.1% loss of the myelinated fibre area were found, respectively, compared to the experimental group which was about 27.1% and 20.1%, respectively, while, in the control group, it was found that at day 28 after operation there was 27.5% loss of the myelinated fibre area compared to the experimental group, where it was only 14.8% loss of the myelinated fibre area. All the data show significant differences (*p* < 0.05) between the control group and experimental group at days 7, 14, 21, and 28 after operation. Thus, this data indicates that EPO supplementation may be related to successful regeneration growth following sciatic nerve crush injury in the rat.

**Figure 8:**
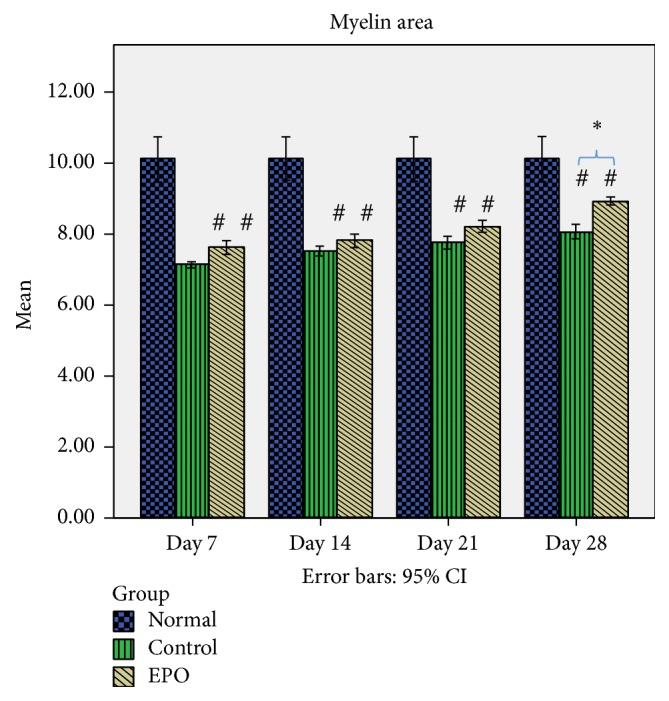
Mean myelin area. The graph shows the value of the mean myelin area (*μ*m^2^) of the sciatic nerve with standard deviations (vertical bars). ^*∗*^*p* < 0.05*, one-way ANOVA when compared at the same time point between control and experimental groups. *^#^*p* < 0.05*, one-way ANOVA when normal group compared with control and experimental groups at each time point.*

Figure 8 shows the mean myelin area of the sciatic nerve in the rats of normal, control, and experimental groups. From the data observed, after the nerve crush injury the size of the myelin area was completely reduced (refer to control group day 7 after operative) compared to the normal group but after that the size of the myelin area increased gradually over time. In the control group, it was found that, at day 7 after operation, there was a significantly higher percentage loss of myelin area (29.5%) compared to the experimental group, where it was only 24.6%. In the control group at days 14 and 21 after operation 25.7% and 23.4% loss of the myelin area were found, respectively, compared to the experimental group which was about 22.9% and 18.7%, respectively, while, in the control group, it was found that at day 28 after operation there was 20.2% loss of the myelin area compared to the experimental group, where it was only 11.6% loss of the myelin area. Thus, this data indicates that EPO supplementation may be related to successful regeneration growth following sciatic nerve crush injury in the rat.

## 4. Discussion

The results of this study indicate that supplementation with EPO is effective for sciatic nerve crush injury in a variety of rodent models especially the rat model. The present study was conducted to determine the potential effect of EPO supplementation on the rate of peripheral nerve regeneration after sciatic nerve injury, through morphological and morphometric analysis of the injured nerve. Oral supplementation of EPO was capable of enhancing nerve regeneration and speeding up the functional recovery of the nerve after the nerve crush injury in the rat.

Based on both the behavioural and histological outcomes, the most significant finding in this study is that EPO possesses potential benefits in improving the toe-spreading reflex (behavioural test) and reducing morphological damage to the sciatic nerve. Many techniques have been developed by researchers and scientists around the world to induce crushes to the sciatic nerve. The most commonly used model is the contusion model. This induces a sciatic nerve injury by weight drop and impactor rod [25]. In this study, we used modified Watchmaker's forceps to induce a crush injury to the sciatic nerve as it is easy to use, is affordable, and has proven to be clinically relevant. Using forceps with different separation distances (calibrated ignition gauge or spacers) at closure point, it is possible to achieve mild, moderate, and severe injuries that can be distinguished histologically or otherwise [26]. However, the forceps induced compression cannot compute the velocity or force delivered at the injury site but it has been shown to be widely used and allows control of injury induction [26]. Our results are similar to the findings of other researchers who have also used forceps to induce crushes in sciatic nerve models [26]. The functional recovery assessment that was used in the present study was the toe-spreading reflex. A simple, precise, and inherently meaningful measure is the return of toe-spreading [27]. In the present study, the findings of functional recovery are supported by histological analysis of the myelin and axon morphology and morphometric analysis. It was found that complete nerve recovery was noted as early as day 9 and as alate as day 15 in experimental group. The mean of complete nerve recovery was at day 12. EPO treatment, therefore, reduced the time taken for complete nerve recovery by 14 days compared to the control group. Furthermore, the result shows that the mean difference between the normal and the EPO group is evidently low compared to the high mean difference between the normal and control group. The implication is that EPO leads to statistically significant TSR recovery at the end experiment (*p* < 0.05). This reduction of 53.8% is higher than the 34.8% reduction observed in the past study [28] where the reduction is statistically significant (*p* < 0.005).

Morphologic and morphometric analysis were used to evaluate the condition of the sciatic nerve after nerve crush injury, and the results were compared to those obtained with the same animals with respect to functional assessment (toe-spreading reflex). In the present study, it should be noted that (in the histological studies) the epineurium was never disrupted in any of the groups, but the perineurium and endoneurium were affected. The histological analysis clearly showed that the myelin was well-arranged in the rats from the normal group but was scattered and irregularly shaped in the rats from the control group. The micrographs of the semithin Toluidine Blue staining showed that the axons and myelin in the experimental group (28 days after operation) had completely recovered or were not markedly different from those of the normal group. At 28 days after operation (experimental group), the axons displayed a rounded structure and the appearance of the myelin sheath became thicker compared to earlier in the nerve crush injury (7 days after operation). The observed myelin sheath changes were similar to previous studies as evaluation studies as this parameter allows for discrimination of the pathological condition of a peripheral nerve from a morphologically and physiologically intact nerve [29–31]. At 7 days after operation, the axons displayed a population of regenerating axons. The size of the axons was small and the myelin sheath was not visible.

From the histological analysis, the mean axon area had significantly decreased from 8.95 *μ*m^2^ in the normal group to 7.20 *μ*m^2^ 7 days after the crush injury (control group). After that, it demonstrated a relative increase 7 days after operation up to 28 days after operation. This finding was comparable with that of a previous study [22] where the value of the normal mean axon area was in the range of 5.38–9.41 *μ*m^2^. Meanwhile, the mean axon diameter followed the same trend as the diameter of the normal axon progressively and significantly decreasing from 2.91 *μ*m (normal group) to 2.24 *μ*m 7 days after the crush injury (control group). This is similar to or not markedly different from the results displayed in previous studies, which indicated that the diameter of the normal axon was in the range of 3.0–3.5 *μ*m and 2.17–2.96 *μ*m [29]. Based on our study, the normal mean myelin area was 10.10 *μ*m^2^. It significantly decreased to 7.12 *μ*m^2^ 7 days after the crush injury. This finding is supported by the results of previous studies [32] where the value of the normal mean myelin area was 12.75 ± 4.37 *μ*m^2^. Similar behaviour was observed for all of the other morphometric parameters (namely, the myelinated fibre area and myelinated axon number), which significantly decreased in all crush injury groups (control and experimental groups) compared to the normal group. However, since most of the differences between the crush injury groups were significant, these findings may be considered normal and indicate that the nerve crush injury produced consistent and virtually similar results [22]. It has been reported that EPO supplementation was responsible for accelerated axonal regrowth in sciatic nerve injured rats [28, 33]. We also believed that EPO can improve Schwann cell proliferation. Schwann cells are responsible for the myelination of peripheral axons. This process occurs when Schwann cell wrap around the axons, creating a myelin sheath from multiple concentric layers of cytoplasmic membrane [4]. The EPO acts via GLA which is a substrate for production of vasodilator prostanoids such as prostacyclin to improve nerve perfusion [34]. Further studies also suggests that cyclooxygenase mediated metabolites such as prostacyclin are vital for EPO's actions and are necessary for maintaining the integrity of sciatic vasa nervorum in rats [35].

## 5. Conclusion

In conclusion, the injury model that has been used in this investigation is an efficient, cost-effective, and ideal way to induce sciatic nerve crush injury in rats. Based on the findings obtained in this study, daily oral EPO supplementation has been shown to enhance the recovery of damaged peripheral nerve. A dose of 6000 mg/day of EPO supplementation helps accelerate nerve regeneration and, therefore, might have a vital role in the therapy of peripheral nerve injury.

## Figures and Tables

**Table 1:** The scoring and grading of the toe-spreading reflex.

Toe-spreading reflex (Gutmann 1942)	
Grade	Clinical symptoms
Degree 0	Absence of any abduction (movement) in any digit.
Degree I	Just visible spreading of the 4th toe alone (or one of the digit)
Degree II	Slight spreading of all three toes
Degree III	Spreading of all three toes less forceful than normal
Degree IV	Full spreading of all three toes (which resembles the unoperated left site)

The toe-spreading reflex is an excellent and sensitive indicator of the onset of nerve and motor neuron recovery [24].

**Table 2:** Pairwise comparisons of the toe-spreading reflex. Measure: MEASURE_1.

(I) group	(J) group	Mean difference (I − J)	Std. error	Sig.^b^	95% confidence interval for difference^b^	
					Lower bound	Upper bound
Normal	Control	1.900^*∗*^	.031	.000	1.820	1.980
	EPO	.400^*∗*^	.031	.000	.320	.480
Control	Normal	−1.900^*∗*^	.031	.000	−1.980	−1.820
	EPO	−1.500^*∗*^	.031	.000	−1.580	−1.420
EPO	Normal	−.400^*∗*^	.031	.000	−.480	−.320
	Control	1.500^*∗*^	.031	.000	1.420	1.580

Based on estimated marginal means. ^*∗*^The mean difference is significant at the .05 level. ^b^Adjustment for multiple comparisons: Bonferroni.

## References

[1] Chiu D. T. W., Ishii C. (1986). Management of peripheral nerve injury. *Orthopedic Clinics of North America*.

[2] Navarro X., Vivó M., Valero-Cabré A. (2007). Neural plasticity after peripheral nerve injury and regeneration. *Progress in Neurobiology*.

[3] Lundborg G. (1988). Intraneural microcirculation. *Orthopedic Clinics of North America*.

[4] Zachodne D. W., Ho L. T. (1990). Endoneural microenvironment and acute nerve crush injury in the rat sciatic nerve. *Brain Research*.

[5] Bagdatoglu C., Saray A., Surucu H. S., Ozturk H., Tamer L. (2002). Effect of trapidil in ischemia/reperfusion injury of peripheral nerves. *Neurosurgery*.

[6] Kerns J. M., Pavkovic I. M., Fakhouri A. J., Wickersham K. L., Freeman J. A. (1987). An experimental implant for applying a DC electrical field to peripheral nerve. *Journal of Neuroscience Methods*.

[7] Wong K.-H., Naidu M., David P. (2011). Peripheral nerve regeneration following crush injury to rat peroneal nerve by aqueous extract of medicinal mushroom *Hericium erinaceus* (Bull.: Fr) Pers. (Aphyllophoromycetideae). *Evidence-Based Complementary and Alternative Medicine*.

[8] Kimura A., Ajiki T., Takeuchi K. (2005). Transmigration of donor cells involved in the sciatic nerve graft. *Transplantation Proceedings*.

[9] Kamada N. (1985). Transplantation tolerance and immunosuppression following liver grafting in rats. *Immunology Today*.

[10] Reichert F., Saada A., Rotshenker S. (1994). Peripheral nerve injury induces Schwann cells to express two macrophages phenotypes: phagocytosis and the galactose-specific lectin MAC-2. *The Journal of Neuroscience*.

[11] Wierzba K., Wańkowicz B., Piekarczyk A., Danysz A. (1984). Cytostatics and immunosuppressive drugs. *Side Effects of Drugs Annual*.

[12] Horrobin D. F. (1992). Nutritional and medical importance of gamma-linolenic acid. *Progress in Lipid Research*.

[13] Gunstone F. D. (1992). Gammar linolenic acid—occurrence and physical and chemical properties. *Progress in Lipid Research*.

[14] Christie W. W. (1999). The analysis of evening primrose oil. *Industrial Crops and Products*.

[15] Yaychuk-Arabie I. (1993). Evening primrose oil: “king’s cure-all”. *Health Naturally*.

[16] Kleijnen J. (1994). Evening primrose oil: currently used in many conditions with little justification. *British Medical Journal*.

[17] Mason P. (1995). Evening primrose oil. *Handbook of Dietary Supplements: Vitamins and Other Health Supplements*.

[18] Horrobin D. F. (1997). Essential fatty acids in the management of impaired nerve function in diabetes. *Diabetes*.

[19] Horrobin D. F. (1983). The role of essential fatty acids and prostaglandins in the premenstrual syndrome. *Journal Reproductive Medicine*.

[20] Jamal G. A., Carmieheal H. (1990). The effect of *γ*-linolenic acid on human diabetic peripheral neuropathy: a double-blind placebo-controlled trial. *Diabetic Medicine*.

[21] Keen H., Payan J., Allawi J. (1993). Treatment of diabetic neuropathy with *γ*-linolenic acid. *Diabetes Care*.

[22] Mazzer P. Y. C. N., Barbieri C. H., Mazzer N., Fazan V. P. S. (2008). Morphologic and morphometric evaluation of experimental acute crush injuries of the sciatic nerve of rats. *Journal of Neuroscience Methods*.

[23] Evening Primrose Oil, 2017, http://www.drugs.com/npp/evening-primrose-oil.html

[24] Gutmann E. (1942). Factors affecting recovery of motor function after nerve lesions. *Journal of Neurology, Neurosurgery & Psychiatry*.

[25] Schmitz H. C., Beer G. M. (2001). The toe-spreading reflex of the rabbit revisited—functional evaluation of complete peroneal nerve lesions. *Laboratory Animals*.

[26] Raducan A., Mirica S., Duicu O. (2013). Morphological and functional aspects of sciatic nerve regeneration after crush injury. *Romanian Journal of Morphology & Embryology*.

[27] Abdullahi D., Annuar A. A., Mohamad M., Aziz I., Sanusi J. (2017). Experimental spinal cord trauma: a review of mechanically induced spinal cord injury in rat models. *Reviews in the Neurosciences*.

[28] Sanusi J. (1996). *The possible of the afferent activity in motoneurone survival after neonatal target deprivation [Ph.D. thesis]*.

[29] Garcia M. L., Rao M. V., Fujimoto J. (2009). Phosphorylation of highly conserved neurofilament medium KSP repeats is not required for myelin-dependent radial axonal growth. *Journal of Neuroscience*.

[30] Samsinah H. H., Junedah S., Norazlin A. K. Effects of Evening Primrose Oil on the rate of nerve regeneration.

[31] Lindemuth R., Ernzerhof C., Schimrigk K. (2002). Comparative morphometry of myelinated nerve fibres in the normal and pathologically altered human sural and tibial nerve. *Clinical Neuropathology*.

[32] Scharpf J., Meirer R., Zielinski M. (2003). A novel technique for peripheral nerve repair. *Laryngoscope*.

[33] Schröder J. M. (1972). Altered ratio between axon diameter and myelin sheath thickness in regenerated nerve fibers. *Brain Research*.

[34] Cameron N. E., Cotter M. A., Robertson S. (1991). Essential fatty acid diet supplementation: effects on peripheral nerve and skeletal muscle function and capillarization in streptozotocin-induced diabetic rats. *Diabetes*.

[35] Cameron N. E., Cotter M. A., Dines K. C., Robertson S., Cox D. (1993). The effects of evening primrose oil on nerve function and capillarization in streptozotocin-induced diabetic rats: modulation by the cyclo-oxygenase inhibitor flurbiprofen. *British Journal of Pharmacology*.

